# Alcohol segment-specific associations between the quality of the parent–child relationship and adolescent alcohol use

**DOI:** 10.1186/1471-2458-14-872

**Published:** 2014-08-23

**Authors:** Jolanda JP Mathijssen, Meriam M Janssen, Marja JH van Bon-Martens, Hans AM van Oers, Elly de Boer, Henk FL Garretsen

**Affiliations:** Tilburg University, TRANZO Department, Academic Collaborative Centre for Public Health Brabant, Post Office Box 90153 5000 LE, 013 4662969 Tilburg, The Netherlands; Regional Public Health Service “Hart voor Brabant”, ‘s-Hertogenbosch, The Netherlands; Trimbos Institute, Netherlands Institute of Mental Health and Addiction, Utrecht, The Netherlands; RIVM, National Institute for Public Health and the Environment, Bilthoven, The Netherlands; Tilburg University, Department TRANZO, Scientific Center for Care and Welfare, Tilburg, The Netherlands

**Keywords:** Audience segmentation, Parent-adolescent relationship, Alcohol-specific rules, Adolescent alcohol use

## Abstract

**Background:**

There is much evidence that parents have an influence on the alcohol use of their children. However, in general the relationship is rather weak. A reason for this small association may be due to the fact that adolescents are a heterogeneous group and that, consequently, the association between the quality of the parent–child relationship and alcohol use varies for diverse subgroups, resulting in an overall small effect. In an earlier study we found five different segments for adolescents regarding their attitude towards alcohol. This article reports on a study into the differences between these segments with respect to the quality of the parent–child relationship and parental attitudes to alcohol. Moreover, we examined segment-specific associations of the quality of the parent–child relationship and alcohol use.

**Methods:**

This study used data from a survey held among adolescents aged 12 to 18. A random sample of 59,073 adolescents was drawn from 67 municipalities in the south of the Netherlands. To assign respondents into one of the five segments, a questionnaire of 28 items concerning alcohol and approval from others from the original segmenting study was included in the internet version. Therefore, only the results of the internet version (N = 12,375 adolescents) were analysed.

**Results:**

Both the quality of the parent–child relationship and the attitude of the parents towards the drinking behaviour of their children differed between the segments. Significant associations were found between the quality of the parent–child relationship and life-time and recent alcohol use and binge drinking. The interaction between the quality of the parent–child relationship and the segments was only significant for binge drinking.

**Conclusions:**

The quality of the parent–child relationship seemed to be most strongly associated with life-time alcohol use, suggesting that parents appear to play the most important role in the prevention of alcohol use. Moreover, the results showed segment-specific associations between the quality of the parent–child relationship and binge drinking, indicating that the role of parents in heavy drinking is different for the various segments.

## Background

There is ample evidence that parents and the quality of the parent–child relationship have an influence on the alcohol use of adolescents. Important factors in this are parental monitoring, communication, parenting behaviours, attitudes and family functioning [1–7]. Positive family dynamics, parental monitoring [8], family bonding [9] and alcohol-specific rules [10–12] serve as protective factors for adolescent alcohol use. A tolerant attitude of parents manifested in e.g. permitting or accepting the alcohol use of their children is associated with frequent alcohol use [13–16], whereas parental disapproval is associated with less alcohol use [17]. Consistent with social control theory [18], a warm and supporting relationship between the parent and child as manifested in e.g. emotional affection, praise and encouragement, is related to less alcohol use [19].

Given the significant role that parents and families play in adolescents’ alcohol use, they are an important target for prevention and intervention. Although reviews of family interventions have suggested consistent effects on the delay of alcohol initiation and the frequency of drinking alcohol, the effects were rather small [20–22]. Moreover, a recent review of longitudinal studies demonstrated only weak evidence for an effect of the parent–child relationship on adolescent alcohol use [23].

One reason for these small effects may be because these interventions targeted adolescents as a homogeneous group in a one-size-fits-all approach, rather than as a heterogeneous group consisting of different subgroups. Possibly, the impact of the parent–child relationship on alcohol use may be stronger for one subgroup compared to another, resulting in an overall small effect. Market research has revealed that in order to meet the needs of the target group, it is important to tailor and fine-tune messages to its different segments [24, 25]. In an earlier study with a different dataset, we examined whether it was possible to segment adolescents according to their values and attitudes towards alcohol. Using latent class analysis, five different segments in a group of 12 to 18-year-old adolescents were distinguished based on five attitude factors (aversion to intoxication, alcohol as norm, need for approval, hedonistic associations, lack of interest in alcohol) [26]. The five segments were designated as: ‘ordinaries’, ‘high spirits’, ‘consciously sobers’, ‘ordinary sobers’ and ‘socials’. The ‘ordinaries’ think alcohol is the norm and they have hedonistic associations with alcohol. The ‘high spirits’ are interested in alcohol, have strong hedonistic associations with alcohol, and have no aversion to intoxication. The ‘consciously sobers’ do not have hedonistic associations with alcohol, are not interested in alcohol and have an aversion to intoxication. ‘Ordinary sobers’ think alcohol is the norm, have hedonistic associations with alcohol, but have an aversion to intoxication and are not interested in alcohol. And finally, the ‘socials’ are interested in alcohol, but they do not think alcohol is the norm, and they have an aversion to intoxication. These five segments also differed in their drinking behaviour independently of socio-demographic variables, with the ‘high spirits’ drinking the most and the ‘consciously sobers’ and ‘ordinary sobers’ the least.

Some studies have revealed that there are gender-specific associations between the parent-adolescent relationship and alcohol use [27, 28]. For example, family conflict was found to be associated with girls’ drinking behaviour but not with boys’ drinking behaviour [27]. However, until now alcohol attitude-based segment-specific associations have never been studied. Therefore, in this study we aim to investigate whether the quality of the parent–child relationship and the attitude(s) of parents regarding the drinking behaviour of their children are different for the five segments. Moreover, we will study whether the association between the quality of the parent–child relationship and alcohol use is different for the distinguished segments.

## Method

### Sampling procedure

This study used data from a survey (the Brabant Youth Health Monitor) held among adolescents aged 12 to 18, collected by three Regional Public Health Services in the Netherlands. A random sample of 59,073 young people aged 12 to 18 was drawn from the Municipal Population Register (MPR) of the 67 municipalities in the province of North-Brabant. The MPR contains the personal data of each member of the Dutch population. From each municipality, depending on its population, at least 550 adolescents were invited to fill out a questionnaire. Parents received a postal invitation with the request to allow their son/daughter to complete the enclosed questionnaire, either on paper or through internet with a personal password. To increase the response rate, two reminders were sent to non-respondents, respectively two and four weeks after the letter of invitation. As an incentive, one in ten young people who completed the online questionnaire received a € 10 cinema voucher.

Respondents were assigned into one of the five segments by means of a 28-item questionnaire on alcohol and approval from others, developed in an earlier study [26]. As these 28 questions were only included in the online version, only the results of the online questionnaire were used.

The survey was approved by the board of directors of the Regional Health Services, and exempted from ethical approval. According to the Dutch Medical Research Involving Human Subjects Act (WMO), these surveys were exempted from ethical approval because they did not subject people to (invasive or bothersome) procedures or required them to follow rules of behaviour.

### Measures

The Brabant Youth Health Monitor was composed using questions from the Dutch local and national youth health monitor [29]. This monitor contains standard questions, which makes it possible to compare results over time and with other regions. Alcohol use was measured with three questions: 1) How often have you drunk alcohol? (life-time alcohol use, 1 = ‘never’ to 13 = ‘20 times or more’); 2) How often did you drink alcohol in the last four weeks? (recent alcohol use, 1 = ‘never’ to 13 = ‘20 times or more’) and 3) How often did you drink five or more glasses of alcohol at a single occasion in the last 4 weeks? (binge drinking, 1 = ‘never’ to 7 = ‘9 times or more’). Since the scores were not measured on a real continuous scale and the distribution was highly skewed, the scores were dichotomized. Only for the drinking adolescents, the attitude of parents regarding the alcohol use of their children was measured with the question How do your parents feel about your alcohol use? ( ‘accept’, ‘think I need to drink less’, ‘discourage’, ‘forbid’, ‘don’t know I use alcohol’, ‘don’t say anything about it’).

The dimension ‘parents’ of the “KIDSCREEN” [30] was part of the Brabant Youth Health Monitor and assesses the quality of the relationship with parents. The KIDSCREEN is a generic questionnaire designed to measure health-related quality of life in children and adolescents aged 8 to 18. The KIDSCREEN was developed in 13 different European countries and tested on a large representative sample of children and adolescents [30]. The version for adolescents (12 to 18-year-olds) was used.

Psychometric properties such as validity and reliability of the KIDSCREEN questionnaire have been assessed in several studies [31, 32] and its cross-cultural comparability and psychometric properties have been found satisfactory. The dimension ‘parents’ consists of six 5-point Likert-type items on a recall period of one week. The scores of these six items were summed to one score with a minimum of 6 and a maximum of 30. The higher the score the better the quality of the parent–child relationship.

As said, respondents were assigned to one of the five segments using a 28-item questionnaire on alcohol and approval from others, developed in the segmenting study [26]. Examples of items are: ‘I would be embarrassed if one of my friends got drunk’, ‘I can imagine that you don’t want to be seen with a soft drink when everyone else is drinking alcohol’, ‘Alcohol makes me think of pleasure and fun’ and ‘Alcohol makes me think: Not for me’.

### Analyses

For this large-scale youth health survey, no simple sampling frame exists (i.e., a single list from which sample members are chosen) for our target population. The population in the province of North-Brabant was divided into groups (i.e. municipalities), and in each municipality a pre-determined number of individuals was sampled. This means that a stratified design was used. In order to adjust for this stratification Complex Samples was used [33]. Complex Samples provides specialised statistics to help correctly compute statistics and their standard errors when working with complex sample designs. Anova analysis was used to investigate whether the quality of the parent–child relationship differed between the segments. Chi-square analysis was used to investigate the differences between the segments in parents’ attitude.

To examine whether the association between the quality of the parent–child relationship and alcohol use (life-time alcohol use, recent alcohol use and binge drinking) differs for the five segments, multiple logistic regression analyses with an interaction effect between the quality of the parent–child relationship and the segment variable were used.

## Results

A total of 28,194 (response rate 48%) 12 to 18-year-olds completed the questionnaire, 56% (n = 15,713) on paper and 44% (n = 12,481) online. For the purpose of this article, only the data of the respondents who filled out the online questionnaire were used. Of these 12,481 respondents, 12,375 completed the 28 items which we needed to assign the adolescents to one of the five segments. For the characteristics of these adolescents see Table 1. There were no statistically significant differences between the adolescents who completed the questionnaire on paper and those who completed the online questionnaire for age, recent alcohol use, and binge drinking. There were, however, differences for sex, ethnic background and ever drunk alcohol. Girls (45% versus 42% for boys), native Dutch (45% versus 39% for immigrants) and respondents who had never drunk alcohol (45% versus 42%) were more likely to fill out the questionnaire online.

**Table 1:** **Characteristics of the respondents** (**N** = **12**,**375**)

Sex	50.5% female
Age	14 years; 11 months
Ethnicity	
Native	86.9%
Western ethnic minority	5.1%
Non-Western ethnic minority	8.0%
Recent alcohol consumption	42.3%
Binge drinking	29.3%
Segments	
Ordinaries	43.0%
High Spirits	20.6%
Consciously Sobers	22.0%
Ordinary Sobers	6.6%
Socials	7.8%
Quality relationship with parents	26.35 (95% CI: 26.25-26.44)
Attitude parents (N =5822)	
Parents accept the alcohol use	58.1%
Parents think their child need to drink less	6.0%
Parents discourage the alcohol use	16.0%
Parents forbid the alcohol use	2.9%
Parents don’t know their child uses alcohol	5.7%
Parents don’t say anything about the alcohol use	11.3%

### Quality of the parent–child relationship and attitude of the parents

Significant differences between the segments were found for both quality of parent-adolescent relationship (F = 66.54 (4) p ≤ .01) and parents’ attitude (Χ^2^ = 365.7 (20) p ≤ .01). Table 2 shows that, compared to the ‘ordinaries’ and ‘high spirits’, the ‘consciously sobers’, ‘ordinary sobers’ and ‘socials’ reported a qualitatively better relationship with their parents.

**Table 2:** **Comparison of the parent**-**adolescent relationship and parents**’ **attitude regarding alcohol use between the five segments**

	1. Ordinaries		2. High spirits		3. Consciously sobers		4. Ordinary sobers		5. Socials		Total
Quality relationship with parents	N = 5360 25.86	^345^	N = 2298 25.63	^345^	N = 2880 27.28	^12^	N = 826 27.42	^12^	N = 1011 27.41	^12^	N = 12375 26.35
Attitude parents	N = 2785		N = 2117		N = 230		N = 86		N = 604		N = 5822
Accept	51%	^25^	64%	^13^	50%	^25^	48%		70%	^13^	58%
Think the adolescent needs to drink less	4%	^235^	10%	^1345^	0%	^12^	1%	^2^	2%	^12^	6%
Discourage	20%	^2^	11%	^134^	27%	^25^	22%	^2^	14%	^3^	16%
Forbid	4%	^5^	2%	^4^	2%		7%	^25^	1%	^14^	3%
Don’t know	8%	^25^	4%	^15^	5%	^5^	5%		1%	^123^	6%
Don’t say anything about it	13%	^2^	9%	^13^	16%	^2^	17%		12%		11%

^12345^ = statistically significant different from other segments at p ≤ .01.

The information on parents’ attitude towards the drinking behaviour of their children obviously applies only to the respondents who drink alcohol (N = 5,822). Parents of ‘high spirits’ and ‘socials’ more often accept the drinking behaviour of their children than parents of ‘ordinaries’ and ‘consciously sobers’. Moreover, parents of ‘high spirits’ think more often that their children need to drink less alcohol than parents of the other segments. Finally, parents of ‘high spirits’ discourage alcohol drinking less often than parents of the other segments, with the exception of parents of the ‘socials’.

### Alcohol use

For both life-time alcohol use, recent alcohol use and binge drinking we ran two models: the first with socio-demographic variables (age, sex, ethnicity), the quality of the parent–child relationship and the segment variable; the second one with the addition of the interaction between the quality of the parent–child relationship and the segment variable. The first model demonstrated that both the quality of the parent–child relationship and the segmentation variable were significantly associated with life-time alcohol use, recent alcohol use and binge drinking independently of socio-demographic variables. The results indicate that the higher the quality of the relationship, the less adolescents had ever drunk alcohol in their lives, the less they had recently drunk alcohol, and the less they had drunk five or more glasses of alcohol at a single occasion in the last 4 weeks .

The second model (see Table 3) showed similar results with only a significant interaction effect for binge drinking. ‘Ordinary sobers’ and ‘ordinaries’ with a better perceived parent-adolescent relationship reported less binge drinking. For the other segments the association between the parent-adolescent relationship and binge drinking was not statistically significant. The Nagelkerke’s R^2^ gives an approximation of the level of explained variance.

**Table 3:** **Association** (**Odds Ratio with 95**% **CI**) **of quality of parent**–**child relationship and segmentation variable with life**-**time alcohol use**, **recent alcohol use and binge drinking**, **adjusted for socio**-**demographic characteristics**

		Life-time alcohol use	Recent alcohol use	Binge drinking
Age	Wald F	1510.67**	1091.60**	1034.00**
		3.14**	2.83**	2.26**
		(2.97-3.33)	(2.66-3.00)	(2.15-2.38)
Sex	Wald F	22.09**	10.47**	5.82*
Boys		Ref	Ref	Ref
Girls		1.48**	1.30**	0.83*
		(1.25-1.73)	(1.11-1.53)	(0.71-0.97)
Ehtnicity	Wald F	55.78**	40.26**	19.02**
Native		Ref	Ref	Ref
Western ethnic minority		0.73	0.52**	0.68**
		(0.46-1.11)	(0.35-0.78)	(0.48-0.97)
Non-Western ethnic minority		0.14**	0.21**	0.35**
		(0.10-0.20)	(0.15-0.30)	(0.25-0.49)
Quality relationship with parents	Wald F	21.46**	8.25**	13.69**
		0.98	0.99	0.97
		(0.91-1.05)	(0.89-1.10)	(0.90-1.06)
Segment	Wald F	3.94**	0.99	2.47*
Ordinaries		Ref	Ref	Ref
High Spirits		6.49**	5.32**	4.69**
		(5.07-8.29)	(4.21-6.72)	(3.93-5.61)
Consciously sobers		0.05**	0.04**	0.03**
		(0.04-0.07)	(0.03-0.06)	(0.02-0.06)
Ordinary sobers		0.13**	0.15**	0.08**
		(0.09-0.18)	(0.10-0.22)	(0.04-0.15)
Socials		1.00	0.96	0.73*
		(0.77-1.30)	(0.72-1.28)	(0.56-0.95)
Interaction between quality of parent–child relationship and segment	Wald F	1.19	1.90	2.86*
Ordinaries		0.91**	0.95*	0.96*
		(0.88-0.93)	(0.92-0.97)	(0.94-0.99)
High Spirits		0.93*	0.99	0.98
		(0.87-0.98)	(0.95-1.03)	(0.94-1.02)
Consciously sobers		0.93	0.93	0.83
		(0.84-1.03)	(0.82-1.06)	(0.84-1.08)
Ordinary sobers		0.90**	0.88**	0.79**
		(0.83-0.97)	(0.81-0.95)	(0.70-0.89)
Socials		1.00	0.99	0.97
		(0.92-1.05)	(0.89-1.10)	(0.90-1.06)
Nagelkerke R^2^		.75	.71	.61

*p ≤ .05.

**p ≤ .01.

## Discussion

There were striking differences between the five segments in the quality of the parent–child relationship and parents’ attitude towards the alcohol use of their children. The ‘consciously sobers’, ‘ordinary sobers’ and ‘socials’ reported a better relationship with their parents than the ‘ordinaries’ and ‘high spirits’. Although the quality of the parent–child relationship for the ‘ordinaries’ and ‘high spirits’ was very similar, there were salient differences between the parents of the two segments. The parents of the ‘ordinaries’ accept the drinking behaviour of their children less often and discourage and forbid it more often than the parents of the ‘high spirits’ do. The attitude of the parents of the ‘consciously sobers’ appears very similar to that of the parents of the ‘ordinaries’. Since strict rules about alcohol seem to prevent adolescents from starting to drink early and progressively more [34–36], the ‘high spirits’ appear to be at the highest risk and the ‘ordinary sobers’ at the lowest risk of heavy drinking.

Our results confirm the social control theory [18], which hypothesizes that the parent–child relationship is associated with adolescent alcohol use or abuse. In our study we found that adolescents who describe the relationship with their parents as more negative reported both more life-time alcohol use, more recent alcohol use and more frequent binge drinking than adolescents who perceived the relationship with their parents as more positive. These associations were statistically significant, independent of socio-demographic characteristics, i.e. sex, age, and ethnicity, and the alcohol segment to which an adolescent belongs.

The relationship with parents seems to be related more strongly to life-time alcohol use than to recent alcohol use and binge drinking, indicating that parents may play an important role in alcohol prevention. The assumption that parents can delay the initiation of adolescent alcohol use is in line with reviews of longitudinal studies [19, 20, 23].

Moreover, we also found significant segment-specific associations between the quality of the parent–child relationship and alcohol use. This means that the role of the quality of the parent–child relationship in adolescent alcohol use seems to differ for the distinguished segments. Significant associations were found between the quality of the parent–child relationship and life-time alcohol use for the ‘ordinaries’, ‘high spirits’ and ‘ordinary sobers’. For recent alcohol use and binge drinking, these associations were found only for the ‘ordinaries’ and the ‘ordinary sobers’. The interaction effect between the segmentation and relation variable was statistically significant only for binge drinking, indicating that especially for heavy drinking the role of parents differs across the various segments.

Although this was obviously not an intervention study, we can formulate some starting points for interventions meant to prevent or reduce children’s alcohol use.

Since parents of the different segments apply different rules for alcohol use, we may conclude that parental rules towards adolescent alcohol use should be part of an intervention. However, although setting alcohol-specific rules has shown to be effective in restraining alcohol use by adolescents (e.g. [10]), restrictive rules seem to be most effective when combined with high quality and frequent communication about alcohol [37]. This seems to indicate that, for instance, teaching parents of ‘high spirits’ to say no to their child using alcohol is not enough if this restrictive rule is not combined with a high quality of the parent–child relationship. Especially for the ‘ordinaries’ and the ‘ordinary sobers’, parents seem to play an important role, in particular in relation to binge drinking. Since this was, to our knowledge, the first study in which segment-specific associations were studied, more research is required to test for the robustness of this association. Moreover, although there is evidence that parenting has an influence on alcohol initiation and on changes in alcohol use [3, 19, 23], the role of the parent–child relationship in this is not clear. Further research is definitely needed to unravel the influence of the parent–child relationship on the initiation into and further course of alcohol use.

Given the strength of the association between the segmentation variable and alcohol use, it is clearly important to also involve adolescents themselves in interventions. This is backed up by findings from a cluster randomised controlled trial comparing the effects of a parent intervention, an adolescent intervention and a combined intervention [38]. Only the combined intervention showed a significant effect on the reduction of weekly and heavy weekly drinking.

In spite of the interesting results, there are some limitations concerning the current study. Firstly, since we could only use the findings of the adolescents who completed the online questionnaire, the studied group is possibly not representative of 12 to 18-year-olds. Secondly, it is important to realise that the quality of the parent–child relationship was only described from the perspective of the adolescent. It is not inconceivable that parents would describe their relationship very differently. However, perceived parenting styles have demonstrated to be related to adolescents’ problem behaviour even after adjustment for sex and family risk to externalising behaviour [39]. Besides, perceived parenting styles were associated with parent as well as teacher-rated problems, confirming that the perception of the adolescents is an important variable. Thirdly, since attitudes seemed to be associated more strongly with alcohol use than the quality of the parent–child relationship [40], it would have been interesting to compare the significance of both aspects. Unfortunately, in our study only adolescents who had recently drunk alcohol completed the question about perceived parental attitudes. In future studies also non-drinking adolescents should be questioned about parental attitudes. Fourthly, the segments are based on a not-yet validated questionnaire. Although the segments were recognised by experts [26] and recent studies seem to demonstrate the usefulness of these different subgroups [41, 42], more research is required to validate the questionnaire. Finally, regarding the cross-sectional character of this study, we cannot rule out the possibility that how adolescents perceive and value alcohol is influenced by their actual alcohol use instead of the other way around. Longitudinal studies are recommended to explore the causality of and the mechanisms explaining the association between alcohol-attitude segments, the parent–child relationship and alcohol use.

## Conclusions

The quality of the parent–child relationship seemed to be most strongly associated with life-time alcohol use, suggesting that parents appear to play the most important role in the prevention of alcohol use. Moreover, the segment-specific associations between the quality of the parent–child relationship and binge drinking, indicate that the role of parents in heavy drinking is different for the various segments. However, further research is necessary to confirm these results.
